# Leçons Theoriques Et Cliniques Sur Les Affections Cutanées Parasitaires

**Published:** 1859-11

**Authors:** 


ART. V.—*Leçons theoriques et cliniques sur les Affections Cutanées Parasitaires, professées* par le DOCTEUR BAZIN, Médecin de l'Hôpital Saint-Louis, Chevalier de la Légion d'Honneur, etc. Rédigées et publiées par M. ALFRED POUQUET, Interne des Hôpitaux. 8vo., pp. 236. Paris: Adrien Delahaye, 1858.

*Theoretical and Clinical Lectures on Parasitic Cutaneous Affections.* By DR. BAZIN, Physician of the Hôpital Saint-Louis, etc. Edited and published by M. ALFRED POUQUET, Interne of the Hospitals.

THE Hôpital Saint-Louis in Paris is devoted mainly to the management of scrofulous and cutaneous cases, and the work before us embraces the theories and results of treatment arrived at by M. Bazin, one of the physicians attached to that institution, in a class of affections of the skin which he considers to be developed by parasitic growths. The author treats of cutaneous affections, (*affections cutanées,*) in contradistinction to cutaneous diseases, (*maladies cutanées,*) and gives his reasons for including, under the common head of parasitic affections, inflammatory conditions of the skin, varied in their appearance and elementary characteristics. A parasitic cutaneous affection (*affection cutanée parasitaire*) he defines as an affection of the skin produced directly by the parasite itself, or symptomatic of a parasitic disease, while by parasitic disease (*maladie parasitaire*) he signifies a peculiar and accidental state of the organism, which exhibits itself as a consequence of the presence of a parasite, whether animal or vegetable, on some part of the body, and which is manifested by a combination of symptoms, lesions, and affections. For example, tinea tondens (*teigne tonsurant*) is a condition which embraces all the expressions of that disease dependent upon the existence of a vegetable parasite, such as erythematous discs, herpes circinatus, eczema circumscriptum, etc.; it is, therefore, a parasitic disease, while each one of its manifestations, herpes circinatus, for example, is a parasitic affection; and the tricophyton is the parasite. In other words, the author wishes to convey the idea that a cutaneous *disease* is a generic term embracing many subdivisions of *affections*, etc. The distinction is one not always made with facility, and, according to this view of M. Bazin, it would be difficult to form a nosological classification that would comprehend all the morbid conditions of the skin.

The subjects embraced in the work are exclusively those to which M. Bazin has devoted special attention at the Hôpital Saint-Louis. He explains the actions of parasites as causes, symptoms, and lesions, or sequences of cutaneous affections, gives a historical sketch of discoveries in regard to vegetable and animal parasites, and the general and special nosography, etiology, therapeutics, etc. of this class of affections. Vegetable parasites are subdivided, according to the portion of skin which they affect, into those which have a preference for the hair and nails, (*végétaux trichophytiques et onychophytiques;*) those which live on the epidermis, (*végétaux épidermophytiques;*) and those on the epithelial surface, (*végétaux épithéliophytiques.*) Animal parasites are divided into two classes, according as they occupy the exterior surface of the body over which they roam at will, or as they are seated in the substance of the epidermis. Discoveries in regard to vegetable parasites are of modern date, only twenty-five or thirty years having elapsed since Schœnlein first described, under the name of Oidium, the vegetable parasite of tinea favosa, which is now known as the Achorion Schœnleinii. Since that time, other interesting discoveries have been made by distinguished microscopists, among whom may especially be mentioned MM. Remak, Bennett, Fuchs, Lebert, Gruby, and Ch. Robin. The knowledge of the existence of animal parasites is of ancient date, but the history of the Acarus, first mentioned by Avenzoar, is of more modern times.

The author sums up briefly the general therapeutics of parasitic cutaneous affections, which is simple and easy, provided the diagnosis is properly made between that class and symptomatic or constitutional eruptions. The three important indications are—First, to destroy the parasite; secondly, to remove the inflammatory affections which are due directly or indirectly to its presence; thirdly, to treat the constitutional eruptions which are often complicated with parasitic affections. The destruction of the parasite, M. Bazin regards as the principal indication upon which a hopeful anticipation of cure may be founded. The parasitic growth is a foreign body, a thorn, which is the cause of all the local irritation; and it is our duty, says M. Bazin, in spite of the intensity of inflammatory symptoms and the extent of surface affected, to extract this thorn, when we may see the symptoms of inflammation extinguished as if by enchantment.

Internal medication has no influence upon these cases, for the preparations employed either do not reach the affected part or are decomposed before they get there. The agents employed externally for the treatment of these affections are classified as *phyticides* and *insecticides*, according to their destructive action on vegetable parasites or animal parasites. Of the former class, M. Bazin prefers the *huile de cade*, (oil of cade,) a liquid tar, derived from the combustion of the wood of the juniperus oxycedrus, and resembling oil of turpentine; corrosive sublimate, and turpeth mineral, or the yellow sulphate of mercury. Of the agents which act most powerfully upon insect parasites, sulphur holds the first place in his opinion, the *huile de cade* and staphisagria or stavesacre being the next most reliable. Anti-parasitic agents may be employed in a semi-solid state, as in the form of ointments, pommades, and liniments, applied by inunction or by gentle or coarse friction; sometimes in the liquid state, as baths, and lotions of different kinds; and occasionally in the gaseous state, as by vapor douches and sulphureous fumigations. The ordinary excipients of *parasiticide* agents are water, oil of sweet almonds, glycerin, spermaceti, and lard; the latter, however, being objectionable from its liability to decomposition by keeping. Where the parasite is deeply seated, as in folliculous parasitic affections, epilation, or the extraction of the hairs by the roots by means of pincers, is recommended in addition to the employment of other agents. Sometimes a single application of an anti-parasitic preparation may suffice; but it may be necessary to repeat it ten, fifteen, or twenty times, the complete destruction of the parasite being the only limit.

M. Bazin asks, rather self-importantly, and not at all flatteringly to his predecessors at Saint-Louis, "Why, before my time, did they not cure scabies (*gale*) at the Hôpital Saint-Louis? Because the value of friction was not appreciated. M. Cazenave, who had charge of the treatment, confined himself to partial friction over the hands, wrists, and feet, and the acari, spread over other parts of the body, being thus untouched, were not slow in reproducing the disease. If sometimes a permanent cure was effected, it was because the patient had rubbed not only his hands and feet, but also every part of his body in which he experienced an itching, and, in this way, had the good fortune to destroy all the animal parasites."

The second indication of treatment is to combat the inflammatory eruption produced either by the parasite or by the application of anti-parasitic medication. To effect this object, emollients, discutients, and antiphlogistics are employed; the most reliable of which are cold cataplasms, pommades medicated with calomel or the oxide of zinc, saturnine lotions, bran or starch baths, cold or vapor douches, and even topical blood-letting by leeches, or the employment of general blood-letting.

The third indication is to treat the various constitutional complications, such as herpetic, syphilitic, and scrofulous eruptions. This influence of parasitic and constitutional cutaneous affections upon one another is occasionally observed. Take, for example, the reciprocal action of syphilis and porrigo decalvans or tinea tondens: the former is often a predisposing cause of the latter, and the vegetable growth found at the roots of the hair in that affection—tricophyton tonsurans—frequently keeps up the syphiloid eruption.

The work of M. Bazin contains peculiar views of pathology, which may not be cordially adopted by other investigators on the same topics, and perhaps occasionally exhibits a more dogmatical and self-lauding spirit than may be absolutely agreeable to those whose theories he demolishes in a few words of disparagement. But it is interesting as a variation from the usual routine classification of the works on cutaneous diseases or cutaneous affections, as authors generally—but not M. Bazin—indiscriminately call them. It is doubtful whether the majority of pathologists will agree with M. Bazin, that the cutaneous affection is the natural sequence of the presence of parasites, but that many of them will prefer the theory that the morbid surface implicated affords a convenient nidus for the propagation and development of the parasitic growth. Under the latter view, the system of medication previously recommended would also be beneficial by setting up a new action in the part, and preventing the growth of the parasite.

